# Surgical prophylaxis in Haydom Lutheran Hospital, Tanzania – learning from a point prevalence survey

**DOI:** 10.1016/j.infpip.2024.100429

**Published:** 2025-01-09

**Authors:** T.J. Schrama, K.J. Vliegenthart-Jongbloed, M. Gemuwang, E.Q. Nuwass

**Affiliations:** a Erasmus University Medical Centre, Department of Internal Medicine, Infectious Diseases Section, Dr. Molewaterplein 40, 3015 GD, Rotterdam, The Netherlands; b Haydom Lutheran Hospital, Haydom, United Republic of Tanzania

**Keywords:** Antibiotic prophylaxis, Surgical prophylaxis, Prevention and control, Drug therapy, Antimicrobial stewardship, Prescription

## Abstract

**Background:**

Antimicrobial resistance (AMR) is a significant global health concern, with improper antibiotic use contributing to its rise. Tanzania initiated an AMR action plan in 2017, but comprehensive surveillance and stewardship efforts remain limited. This study focused on evaluating antibiotic use, particularly surgical prophylaxis, in a rural Tanzanian hospital.

**Methods:**

The study was conducted at Haydom Lutheran Hospital in Tanzania in May 2023, using a cross-sectional point prevalence survey. Antibiotic use in all patients admitted for >24 h and those undergoing surgery was recorded, including type, dose, indication and duration. Quality indicators for surgical prophylaxis were assessed.

**Results:**

Among 199 inpatients, 55% received antibiotics, with surgical prophylaxis accounting for 23% of prescriptions. Notably, none of the patients who received surgical prophylaxis received a single-dose regimen, and 67% exceeded the recommended 24-h duration. A combination of ampicillin-cloxacillin plus metronidazole was the most commonly prescribed combination for surgical prophylaxis (41% of prescriptions). Thirty-three percent of the antibiotics prescribed for surgical prophylaxis were classified as ‘Not recommended’ by the World Health Organization. Furthermore, 90% of surgical prophylaxis prescriptions lacked documented rationale, and 83% of prescriptions lacked stop/review dates in medical records.

**Conclusion:**

This study reveals a high prevalence of prolonged antibiotic use for surgical prophylaxis, frequent use of antibiotics classified as ‘Not recommended’, and a lack of adequate documentation, which deviates from international standards. These practices highlight the urgent need for contextualized national guidelines, large-scale implementation projects of evidence-based interventions, and local initiatives in antibiotic stewardship, particularly in low-resource settings.

## Introduction

Antimicrobial resistance (AMR) caused an estimated 4.95 million deaths globally in 2019, with the Eastern Sub-Saharan African region ranking second [1]. A key driver is the improper and excessive use of antimicrobial medications [2]. Tanzania launched a national action plan in 2017 to combat AMR by implementing antimicrobial stewardship [3]. Goals included enhancing patient outcomes, reducing microbial resistance, and reducing the spread of infections caused by multi-drug-resistant organisms [4]. Although efforts have been made in data collection, there remains a lack of publications on comprehensive national or regional surveillance data [5,6].

According to the World Health Organization (WHO) guidelines, implementing antimicrobial stewardship within healthcare facilities starts with evaluating prescription practices to identify areas for improving antibiotic use [7,8]. Point prevalence surveys (PPSs) conducted in other regions of Tanzania revealed that 30–62% of inpatients received antimicrobials, mainly from the WHO ‘Access’ group [[9], [10], [11], [12]]. Of particular concern was the prolonged use of antibiotics for surgical prophylaxis.

Internationally, numerous organizations have established guidelines for the use of surgical prophylaxis, including WHO, the Infectious Diseases Society of America, and the Surgical Infection Society [[13], [14], [15]]. Surgical interventions are stratified by infection risk based on location, length of the procedure, and wound classification to determine the need for prophylaxis. Consistent with these guidelines, if prophylaxis is indicated, the initial administration of antibiotics should occur within 60 min of the first incision, with prophylaxis preferably limited to 1 day [[13], [14], [15]]. For most surgical indications, antibiotic prophylaxis for >24 h does not prevent the development of postoperative infections compared with surgical prophylaxis for ≤24 h, but increases the risk of antimicrobial resistance and side effects [13]. This recommendation is valid for high-income countries as well as developing countries [16]. Despite this, a recent study in four low- and middle-income countries found that 92.9% of patients received antibiotics for prophylaxis after surgery, and 27.7% received antibiotics for ≥24 h [17].

In six referral hospitals in Tanzania, over half of antibiotic prescriptions were for surgical prophylaxis [9], and a teaching hospital reported that 97% exceeded WHO's recommended single-dose regimen [11]. However, in Tanzania, AMR surveillance is mainly conducted in urban areas, and data from rural areas remain scarce [18].

As such, the AMR team at Haydom Lutheran Hospital conducted a facility-based PPS, focusing on the nature and duration of surgical prophylaxis, to identify barriers and opportunities for improvement within the study hospital and Tanzania's broader healthcare context.

## Methods

### Setting

Haydom Lutheran Hospital, located in the rural Manyara region of Tanzania, is a 315-bed church-owned referral hospital serving 5.7 million people. The hospital encompasses inpatient wards for medical, paediatrics, surgery, orthopaedics, and gynaecology and obstetrics. Annually, the hospital conducts approximately 3000 operations. The hospital utilizes a digital patient filing system in English, allowing drug prescriptions to be issued electronically at the patient's bedside using mobile phones or tablets.

### Global PPS of antimicrobial consumption and resistance

This study followed the open access ‘Global point prevalence survey of antimicrobial consumption and resistance’ methodology protocol (version April 2023; www.global-pps.com), herafter referred to as ‘Global-PPS’ [8]. The Global-PPS is a completely anonymous audit of local antibiotic prescribing practices, coordinated at the University of Antwerp, Belgium since 2014. The aim of the Global-PPS is to survey performance indicators, identify targets for improving antimicrobial prescribing, and assist in designing hospital interventions to promote prudent antibiotic use.

### Data collection and analysis

On Tuesday 23 May 2023, a data collection team (two intern doctors, two international students and an infectious diseases consultant) conducted a cross-sectional hospital-based study. All inpatients, regardless of age, who were present on the ward at 8:00 AM were included in the study. Patients in temporary units, such as the emergency department and renal dialysis unit, were excluded, as were discharged patients who remained on the wards while awaiting payment. In consultation with the Global-PPS support team, a Tuesday was chosen to avoid reflecting the prescribing behaviour over the weekend, when no scheduled surgeries were performed.

The Global-PPS web application was used for data collection [8]. This application facilitated the entry of anonymized and coded data on the total number of patients on each ward, as well as those receiving antibiotic therapy in the 24 h prior to inclusion. Detailed information regarding the type, dose, route of administration, indication, and duration of antibiotic use was documented.

In accordance with the Global-PPS framework, criteria for evaluating the quality indicators of prophylactic prescriptions included adherence to guidelines, documented reasons for prescription, and the presence of stop/review dates. Compliance with guidelines was verified against the 2021 ‘Standard Treatment Guidelines and National Essential Medicines List for Tanzania Mainland’ (STG) [19]. In instances of uncertainty, the infectious diseases specialist, integral to the team, made the final decision.

Data analysis was conducted using Microsoft Excel (2019), encompassing descriptive statistical analyses of key quality indicators, including the prevalence of overall inpatient antibiotic usage, indication and duration. Antibiotics were classified as ‘Access’, ‘Watch’, ‘Reserve’ or ‘Not recommended’ using the 2023 WHO AWaRe classification list [12,20]. Definitions of AWaRe categories can be found in Table I.

**Table I:** AWaRe categories, definitions and list of antimicrobial drugs used for surgical prophylaxis in this point prevalence study

Category	Definition	Antibiotic
Access	Lower resistance risk; first- and second-line treatments; inexpensive and safe	Ampicillin, gentamicin, metronidazole
Watch	Broad-spectrum; higher resistance risk; use if ‘Access’ options are not suitable	Ceftriaxone, ciprofloxacin
Reserve	Last-line options for multi-drug-resistant organisms	-
Not recommended	Antibiotics whose use is not advised	Ampicillin-cloxacillin

Definitions and classification of antibiotics are adopted from the World Health Organization's AWaRe antibiotic book [20] and AWaRe classification list [12].

### Ethical approval

Permission to conduct the study was granted by the Managing Medical Director. Informed consent was not applicable as there was no direct patient contact and all data were anonymized. Each hospital participating in the Global-PPS remains the owner of its own data at all times.

## Results

### Patient characteristics

At 8.00 am on the day of the PPS, 199 patients had been admitted to the hospital (Table II). Overall, antimicrobial drugs were prescribed to 55% (110/199) of patients, with rates of 39% (13/33) on the obstetric ward, 41% (27/66) on the surgical ward, 61% (20/33) on the medical ward, and 75% (50/67) on the paediatric ward. Most patients were male (56%) and adult (55%). Of the patients who were prescribed antibiotics, 66.4% had two prescriptions running simultaneously and 7.3% had three prescriptions or more running simultaneously (Table II).

**Table II:** Hospital and patient characteristics on the day of the point prevalence study

Admitted patients	199
Patients on antimicrobial drugs	110 (55.3%)
Gender	
Male	61 (55.5%)
Female	49 (44.5%)
Age group	
Neonate (≤1 month)	22 (20.0%)
Child (>1 month–≤17 years)	28 (25.5%)
Adult (≥18 years)	60 (54.5%)
Number of prescriptions	
1	29 (26.3%)
2^a^	73 (66.4%)
≥3^a^	8 (7.3%)

Values are presented as *N* (%).

a All antimicrobial prescriptions regardless of whether they were for the same diagnosis or multiple concurrent infections.

### Pattern of antimicrobial prescribing

In total, 201 prescriptions were recorded in the PPS, of which 68.7% (138/201) were for therapeutic use (Table III). The most common indications for therapeutic use were pneumonia, skin and soft tissue infections, and sepsis (Table IV). Antimicrobial prescriptions for prophylaxis accounted for 30.8% (62/201) of all prescriptions, and surgical prophylaxis was the most common reason [22.8% of all prescriptions (46/201)] (Table III). Surgical prophylaxis consisted of the following indications: abdominal surgery [17% (8/46)], orthopaedic surgery [37% (17/46)], and obstetric/gynaecological surgery [46% (21/46)]. In total, 27 patients received surgical prophylaxis. None of these patients received single-dose prophylaxis; 33% received multiple doses within 24 h and 67% received multiple doses for >24 h (Table IV) The majority of patients (41%) received a combination of ampicillin-cloxacillin plus metronidazole as surgical prophylaxis, most of whom were admitted for obstetric and gynaecological surgery (Table V). On this ward, this combination was used after gynaecological surgery, caesarean sections, and episiotomies performed for vaginal deliveries.

**Table III:** Indication of the prescribed antimicrobial drug: therapeutic or prophylactic use (*N*=201)

Therapeutic use	138 (68.7%)
Community-acquired infection	124 (89.9%)
Healthcare-associated infection	14 (10.1%)
Prophylactic use	62 (30.8%)
Medical prophylaxis	16 (25.8%)
Surgical prophylaxis	46 (74.2%)
One dose	0 (0.0%)
1 day	15 (32.6%)
>1 day	31 (67.4%)
Unknown	1 (0.5%)

Values are presented as *N* (%).

**Table IV:** Distribution of prescribed antimicrobial treatment based on various clinical indications (*N*=201)

Pneumonia or lower respiratory tract infections	65 (32.2%)
Prophylaxis for obstetric or gynaecological surgery	21 (10.4%)
Prophylaxis for plastic or orthopaedic surgery	14 (7.0%)
Medical prophylaxis for neonates with risk factors	14 (7.0%)
Skin and soft tissue infections	13 (6.5%)
Sepsis of any origin	12 (6.0%)
Gastrointestinal infections	9 (4.5%)
Pulmonary tuberculosis	8 (4.0%)
Prophylaxis for gastrointestinal surgery	8 (4.0%)
Obstetric or gynaecological infections	7 (3.5%)
Other	30 (14.9%)

Values are presented as *N* (%).

**Table V:** Prescribed combinations of antimicrobial drugs in patients receiving surgical prophylaxis

	Overall (*N*=27)	Surgical (*N*=18)	Obstetrics and gynaecology (*N*=11)
Ampicillin-cloxacillin plus metronidazole	11 (41%)	3 (17%)	8 (73%)
Ampicillin-cloxacillin	4 (15%)	4 (22%)	-
Ceftriaxone plus gentamicin	4 (15%)	4 (22%)	-
Ceftriaxone plus metronidazole	3 (11%)	2 (11%)	1 (9%)
Ceftriaxone	2 (7%)	2 (11%)	-
Ciprofloxacin	2 (7%)	2 (11%)	-
Ampicillin plus metronidazole	1 (4%)	1 (6%)	1 (9%)

Values are presented as *N* (%).

### Quality indicators of surgical prophylaxis prescriptions

Based on the WHO AWaRe classification, surgical prophylaxis prescriptions were categorized as ‘Access’ [43% (20/46)], ‘Watch’ [24% (11/46)], ‘Reserve’ [0% (0/46)] or ‘Not recommended’ [33% (15/46)]. Ceftriaxone (9/11) and ciprofloxacin (2/11) were among the antimicrobials classified in the ‘Watch’ group. All prescriptions categorized as ‘Not recommended’ consisted of the fixed combination ampicillin-cloxacillin, administered either orally as 250/250 mg or intravenously as 500/500 mg, three times daily. Evaluating adherence to guidelines presented challenges, as 85% of prophylactic prescriptions lacked corresponding guidance in the national guidelines (Table VI). In instances where guidance existed, the prophylaxis was deemed inappropriate or unnecessary. Additionally, 90% of prescriptions lacked documented justifications for their necessity, and the majority did not specify stop/review dates.

**Table VI:** Quality indicators of surgical prophylaxis prescriptions (*N*=46)

Reason for prescription in notes	
Yes	5 (10.9%)
No	41 (90.1%)
Guideline adherence	
Yes	0 (0.0%)
No	7 (15.2%)
Not available	39 (84.8%)
Stop/review date in notes	
Yes	8 (17.4%)
No	38 (82.6%)

Values are presented as *N* (%).

## Discussion

### Antibiotic use in comparison with other studies

In this PPS, 55% of patients were prescribed at least one antibiotic on the day of the survey, which is comparable with prevalence rates reported from Tanzania (44–66%) [9,11,21] and other African countries (28–75%) [10,22]. As expected, this was higher than antibiotic prescribing in hospitals located in Western Europe (28%) and North America (38%) [22]. In the present study, surgical prophylaxis contributed to a total of 23% of all antibiotic prescriptions, ranking second only after pneumonia (32%). This is in the lower range of percentages of antibiotics prescribed for surgical prophylaxis observed in six Tanzanian referral hospitals (30%) and three hospitals in North-Eastern Tanzania (24–35%), and is definitely lower than the percentage observed in a study comprising 17 hospitals across Ghana, Uganda, Zambia and Tanzania (Moshi), where surgical prophylaxis was the primary reason for antibiotic prescription [10]. An outlier in this regard is the national hospital in the capital, where surgical prophylaxis accounted for only 3% of prescriptions, surpassed not only by hospital-acquired infection (6%) but also by an exceptionally high number of undocumented indications (36%) [21]. To put this into perspective, the percentage of antibiotic prescriptions for surgical prophylaxis in Western Europe and North America is considerably lower at 12% and 8.6%, respectively [22].

The reasons for the lower proportion of antibiotics prescribed for surgical prophylaxis in Haydom Lutheran Hospital compared with the other surveyed hospitals in Tanzania remain unclear. Whereas data from other hospitals all originate from an urban setting, Haydom Lutheran Hospital is situated in a more rural area (Supplementary Table I). It is hypothesized that this could result in a higher share of infectious diseases, such as pneumonia, requiring antibiotic treatment. Moreover, the number and complexity of surgical interventions could be lower. Although lower prescriptions could be the result of a more effective stewardship programme, this seems unlikely. The six referral hospitals are all national AMR intervention sites, with training and AMR surveillance. The hospital in Moshi is supported by international research collaborations as well as strong diagnostic facilities.

### Surgical prophylaxis indication and antibiotic choice

Although more than one-fifth of prescriptions were for surgical prophylaxis, the Tanzanian STG [19] lack specific recommendations concerning surgical prophylaxis, encompassing indications, antibiotic selection, and duration. Additionally, there are no locally available guidelines on this matter.

Despite being the first choice internationally, cefazolin and cefuroxime were not available for prescription at Haydom Lutheran Hospital during this PPS. In this study, the fixed drug combination with ampicillin and cloxacillin was the most prescribed prophylaxis, which is consistent with findings from a previous study [9]. However, this combination is not recommended by WHO for either prophylaxis or treatment [12,23]. The ampicillin component is not recommended to cover *Staphylococcus aureus* due to universal resistance [24]. The addition of metronidazole was also frequently observed in this PPS. Anaerobic coverage is only recommended in the case of surgery involving intestinal, and selected gynaecological or ear–nose–throat procedures [25]. This is usually done by the addition of metronidazole to cefazolin, instead of switching to broad-spectrum antibiotics (e.g. amoxicillin-clavulanic acid) which target Gram-negative bacteria unnecessarily [14]. The routine addition of metronidazole to amoxicillin-clavulanic acid is often referred to as ‘double anaerobic coverage’. Despite the fact that this practice still appears in the Tanzanian STG [19], it was not observed in this or earlier studies [9]. Preventing double anaerobic coverage aligns with antimicrobial stewardship goals, as it is correlated with heightened risk of drug resistance, adverse reactions, and increased hospital costs [26].

### Duration of surgical prophylaxis

This study found a significant gap in adherence to the WHO antimicrobial stewardship guidelines [27], with 90% of surgical prophylaxis prescriptions lacking documented justifications, and 83% lacking stop/review dates in medical records. Moreover, two-thirds of surgical prophylaxis prescriptions lasted for >1 day, which is an enormous proportion. However, in earlier studies, the proportion almost reached 100% [10,11], with the exception of Mawenzi regional referral hospital (77%) [11]. This situation reflects inappropriate, unassessed, prolonged antibiotic administration, potentially exacerbating AMR.

It remains unclear whether lack of knowledge, lack of awareness, or local behavioural patterns contribute to inappropriate prophylaxis prescribing practices. There are no guidelines other than the Tanzanian STG, which do not include recommendations on the duration of prophylaxis. At Haydom Lutheran Hospital, antibiotics are prescribed exclusively by medical doctors. However, a significant portion of the prescribing staff comprises intern doctors, who conduct most ward rounds under the supervision of a registrar and/or specialist.

### Contextual challenges

Local circumstances pose challenges to the implementation of a surgical prophylaxis policy aligned with international guidelines. In the authors' experience, local healthcare providers may perceive that international guidelines cannot be extrapolated to their setting. They tend to prioritize extended prophylaxis or proactive treatment, rather than adopting a ‘wait-and-see’ approach [28]. The following factors are from the authors' own experience:

(1) Absence of localized or national guidelines specifically outlining the recommended timing, duration and choice of agents for surgical prophylaxis in Tanzania.
(2) Non-availability of key antimicrobial agents such as cefazolin and flucloxacillin in certain regions, like Haydom, which hampers the ability to follow international recommendations that include these antibiotics.
(3) Limited availability of microbiological resources restricts healthcare providers' access to vital information concerning local bacterial prevalence and resistance patterns. This includes challenges such as the unknown prevalence of meticillin-resistant *S. aureus*, the inability to culture anaerobic bacteria, and lack of structured data on the involvement and susceptibility of Gram-negative micro-organisms in surgical site infections. This lack of data often leads to the use of broad-spectrum antibiotics for prophylaxis to mitigate the risk of severe infections that are diffcult to treat.
(4) Financial constraints, which include limitations on prolonged hospital stays, re-operations, access to microbiological culturing, reserve antibiotics, and imaging modalities such as computed tomography scans.
(5) The learning approach in African countries differs from that in Western countries. Young doctors in Africa primarily learn through hands-on experience and by observing and learning from specialists. Hierarchy plays a significant role, and prophylactic behaviour is often adopted by interns through echoing the practices of their senior colleagues [29].
(6) Sanitation conditions at patients' homes are significantly inadequate, primarily attributable to traditional housing, overcrowding, limited access to clean water, and residing closely with animals.
(7) Follow-up is challenging, as patients face financial constraints and difficulties with transportation, hindering regular visits to the hospital.
(8) While the Tanzanian infection prevention and control guidelines are well crafted and comprehensive, there is a substantial gap between these guidelines and their practical implementation due to severe infrastructural limitations and resource constraints.

### Recommendations for improvement

To improve prophylactic antibiotic use, a multi-faceted approach is essential, as outlined in the review by Wu *et al.* [30]. Based on the findings of this study, the following four priorities are proposed. Firstly, incorporating explicit prophylaxis guidelines into the Tanzanian STG. It should incorporate perioperative prophylactic indications, antibiotic choice, dose, timing and duration. Prescribers are reported to be more receptive to concise guidelines that clearly outline the appropriate antibiotics for common infections [30]. Ideally, prophylaxis guidelines should prioritize ensuring the availability of the necessary drugs, rather than using available drugs for prophylaxis. Secondly, to further roll out evidence-based interventions, such as the successful study by Allegranzi *et al.*, reducing surgical site infections in five African hospitals using a multi-modal infection control and patient safety intervention [31]. Collection of implementation data is pivotal. International and national leadership, together with local ownership is needed to finance and organize large-scale quality improvement projects, conducted explicitly in real-life settings with limited research support from outside. Thirdly, the requirement for surveillance data is crucial. This includes not just national and local antibiotic susceptibility patterns, but also the rates of postoperative infections. This requires access to diagnostic medical microbiology facilities. Fourthly, hospital managements should encourage local initiatives, including education and training that emphasizes antibiotic prescription, activities supporting general infection prevention measures, and the influence of informed local role models. Additionally, implementing structural repetition of a PPS can offer valuable insights into current practices, aiding in the identification of areas for improvement [32]. Surveys can form the basis for targeted interventions and continuous monitoring of the effectiveness of implemented changes. Through these multi-faceted and synergistic interventions, the rational use of prophylactic antibiotics can be enhanced significantly, aligning with both local needs and international standards.

### Limitations

Limitations of this study include the cross-sectional design of the PPS, which provided a snapshot of the situation. Additionally, the day of the PPS influenced the duration of surgical prophylaxis (i.e. some patients were assessed <24 h after surgery but received antibiotics for >24 h). This could lead to underestimation of the duration of prophylaxis. Furthermore, local prescribers may have been pre-informed and discontinued antibiotics. The hospital management approved the conduction of the PPS, but the hospital staff were not informed of the date. However, the staff designing and conducting the PPS included the only surgical registrar (MG), one of two internists (KV) and one of the three surgeons in the surgery department (EN). Due to the size of the hospital, it would have been unethical not to work on the days preceding the survey. Although the authors attempted to maintain their usual conduct, it is possible that the upcoming PPS influenced their actions. It is worth noting that the indications for some antibiotics may have differed from what was assumed, due to incomplete medical records or inadequate documentation of patient conditions. Certain instances of prolonged prophylaxis may, in fact, involve the treatment of peritonitis or active infections. Inadequate record-keeping poses challenges for comprehensive analysis of the outcomes. Despite these limitations, the percentage of antibiotic usage and the types of antibiotics used align with previous studies, as described.

In conclusion, this PPS at Haydom Lutheran Hospital in rural Tanzania showed that a significant proportion of patients received surgical antibiotic prophylaxis, deviating from international guidelines. Notably, no patients received single-dose prophylaxis, and two-thirds of cases exceeded the recommended 24-h duration. These practices underscore the pressing need for contextualized national guidelines, large-scale implementation projects of evidence-based interventions, and local initiatives in antibiotic stewardship, particularly in low-resource settings. This study highlights the challenges of implementing global standards in such contexts, where the absence of local guidelines, resource constraints, and educational disparities influence antibiotic prescribing practices significantly.

## Author contributions

Thijs Schrama: data collection and curation, writing – original draft. Klaske Vliegenthart: conceptualization, methodology, supervision, data collection, writing – original draft. Martin Gemuwang: data collection, reviewing and editing. Emmanuel Nuwass: conceptualization, methodology, supervision, review and editing.

## Disclosure

During the preparation of this work, the authors used ChatGPT in order to rephrase and optimize language. After using this tool, the authors reviewed and edited the content as needed, and take full responsibility for the content of the publication.

## Funding sources

This study was conducted without external funding. The Haydom Lutheran Hospital management approved the allocation of time for individuals involved in performing the PPS.

## Conflict of interest statement

None declared.
