# Supplementary material for: Surgical prophylaxis in Haydom Lutheran Hospital, Tanzania – learning from a point prevalence survey
Source: Infect Prev Pract. 2025 Jan 9;7(1):100429. doi: 10.1016/j.infpip.2024.100429 (PMC11804529; doi:10.1016/j.infpip.2024.100429)
Supplement: Multimedia component 1 [file mmc1.docx]

| **Supplementary Table I**  Overview of Tanzanian cities, population density, and hospitals involved in point prevalence studies (PPS) in Tanzania | | | |
| --- | --- | --- | --- |
|  | **Population (town)** | **Population density/km (region)** | **Hospitals included in antibiotic PPS in Tanzania** |
| **Dar Es Salaam, Dar Es Salaam** | 5.383.728 | 3.865 | Temeke RRH [9]  Muhumbili National Hospital [21] |
| **Mwanza, Mwanza** | 1.104.521 | 391 | Sekou Toure RRH [9] |
| **Dodoma, Dodoma** | 765.179 | 75 | Benjamin Mkapa ZRH [9] |
| **Mbeya,  Mbeya** | 541.603 | 62 | Mbeya ZRH [9] |
| **Kigoma, Kigoma** | 232.388 | 67 | Maweni RRH [9] |
| **Moshi, Kilimanjaro** | 221.733 | 141 | Kilimanjaro Christian Medical Centre RRH [11]  Mawenzi RRH [11]  St. Joseph Hospital [11] |
| **Bukoba, Kagera** | 144.938 | 118 | Bukoba RRH [9] |
| **Haydom, Manyara** | 30.631 | 43 | Haydom Lutheran Hospital RRH |
| RRH, regional referral hospital; ZRH, zonal referral hospital.  Data on population numbers accessed via [33]. | | | |

Reference

[9] Seni J, Mapunjo SG, Wittenauer R, Valimba R, Stergachis A, Werth BJ, et al. Antimicrobial use across six referral hospitals in Tanzania: a point prevalence survey. BMJ Open 2020;10:e042819.

[11] Horumpende PG, Mshana SE, Mouw EF, Mmbaga BT, Chilongola JO, de Mast Q. Point prevalence survey of antimicrobial use in three hospitals in North-Eastern Tanzania. Antimicrob Resist Infect Control 2020;9:149.

[21] Katyali D, Kawau G, Blomberg B, Manyahi J. Antibiotic use at atertiary hospital in Tanzania: findings from a point prevalence survey. Antimicrob Resist Infect Control 2023;12:112.

[33] National Bureau of Statistics Tanzania. Matokea ya sensa ya sita. Census data 2022. Available at: https://www.citypopulation.de/en/tanzania/cities/ [last accessed July 2024].
